# Long and short range order structural analysis of *In-situ* formed biphasic calcium phosphates

**DOI:** 10.1186/s40824-015-0036-0

**Published:** 2015-12-31

**Authors:** Dong-Hyun Kim, Kyu-Hong Hwang, Ju Dong Lee, Hong-Chae Park, Seog-Young Yoon

**Affiliations:** School of Materials Science and Engineering, Pusan National University, Busan, 609-735 Republic of Korea; School of Nano and Advanced Materials, Gyeongsang National University, Jinju, Gyeongnam 660-701 Republic of Korea; Korea Institute of Industrial Technology, Busan, 618-230 Republic of Korea

**Keywords:** Biphasic calcium phosphate, Rietveld refinement, Raman spectroscopy

## Abstract

**Background:**

Biphasic calcium phosphates (BCP) have attracted considerable attention as a bone graft substitute. In this study, BCP were prepared by aqueous co-precipitation and calcination method. The crystal phases of *in-situ* formed BCP consisting of hydroxyapatite (HAp) and β-tricalcium phosphate (β-TCP) were controlled by the degree of calcium deficiency of precursors. The long and short range order structures of biphasic mixtures was investigated using Rietveld refinement technique and high resolution Raman spectroscopy. The refined structural parameters of *in-situ* formed BCP confirmed that all the investigated structures have crystallized in the corresponding hexagonal (space group P63/m) and rhombohedral (space group R3c) structures.

**Results:**

The crystal phases, Ca/P molar ratio, and lattice parameters of *in-situ* formed BCP consisting of HAp and β-TCP were controlled by the degree of calcium deficiency of calcium phosphate precursors. The significant short range order structural change of BCP was determined by Raman analysis.

**Conclusions:**

The long and short range order structural changes of *in-situ* formed BCP might be due to the coexistence of β-TCP and HAp crystal phases.

**Electronic supplementary material:**

The online version of this article (doi:10.1186/s40824-015-0036-0) contains supplementary material, which is available to authorized users.

## Background

Biphasic calcium phosphates (BCP) have attracted considerable attention as a bone graft substitute [1–4]. Generally, BCP consisting of biocompatible hydroxyapatite (HAp) and biodegradable β-tricalcium phosphate (β-TCP) have better bio-resorbability and osseointegration than the individual HAp or β-TCP components because of their different dissolution behaviors under *in vitro* and *in vivo* biological conditions [5, 6].

Several researchers have recently attempted to develop BCP comprising HAp and β-TCP as well using various synthetic routes, such as the blending of different calcium phosphates in solid state reactions, precipitation, liquid mix techniques, treatment of natural bone, spray pyrolysis, and microwave and combustion processing [7–12]. For examples, BCP are usually produced either by the mechanical mixing and sintering of monophasic HAp/β-TCP powder mixtures (i.e., ex-situ formation) or by calcining of a single phase calcium deficient hydroxyapatite (CDHA) powders (i.e., *in-situ* formation). In the case of CDHA, It is well known that BCP and bi-phases can be obtained and controlled through *in-situ* process of heating CDHA (Ca/P = 1.5 ~ 1.67) above 700 °C according to the Equation (1):

1 $$ \mathrm{C}{\mathrm{a}}_{10\hbox{-} \mathrm{x}}{\left(\mathrm{H}\mathrm{P}{\mathrm{O}}_4\right)}_{\mathrm{x}}{\left(\mathrm{P}{\mathrm{O}}_4\right)}_{6\hbox{-} \mathrm{x}}{\left(\mathrm{O}\mathrm{H}\right)}_{2\hbox{-} \mathrm{x}}\circledR\ \left(1\hbox{-} \times \right)\mathrm{C}{\mathrm{a}}_{10}{\left(\mathrm{P}{\mathrm{O}}_4\right)}_6{\left(\mathrm{O}\mathrm{H}\right)}_2 + 3\times \mathrm{C}{\mathrm{a}}_3\left(\mathrm{P}{\mathrm{O}}_4\right) + \mathrm{x}{\mathrm{H}}_2\mathrm{O} $$

In addition, the *in-situ* formation method of BCP can be also applied to various studies of ionic substitutions, biopolymer/calcium phosphate composites, local drug delivery system, and porous scaffolds [13–16]. Because the studies of various attempts for BCP still need to be focused in order to optimize the biological performances. Consequently, fundamental efforts to improve the biological response of *in-situ* formed BCP have recently based on studies of various biphasic controls of HAp/TCP ratios.

The research for crystal structure of *in-situ* formed BCP has been the subject of specific interest owing to its essential biological role in the comprehension for coexistence of two crystal phases. Despite having the similar elemental composition, HAp (Ca_10_(PO_4_)_6_(OH)_2_) and β-TCP (Ca_3_(PO_4_)_2_) differ considerably in their crystal system. For example, crystal system of β-TCP has generally a rhombohedral (space group R3c) or hexagonal structure (space group R3c). HAp has a hexagonal (space group P63/m) or monoclinic structure (space group P21/b). Such different crystallographic forms of HAp and β-TCP can be performed the different biological properties related to biodegradation and dissolution rate. Therefore, if a crystal system of BCP can be controlled and varied by *in-situ* formed bi-phases, it can suggest a new paradigm of bioceramic applications for improve biological properties. In this study, the ability to clearly identify an individual crystal structure of *in-situ* formed BCP demonstrate in the long and short range order structural analysis using the Rietveld refinement of X-ray diffraction (XRD) spectra and high resolution Raman spectroscopy.

## Methods

β-TCP, HAp, and BCP powders were synthesized by the co-precipitation and calcination process. Firstly, an appropriate amount (Ca/P molar ratio 1.5 ~ 1.67) of calcium nitrate tetrahydrate (Ca(NO_3_)_2_°4H_2_O, Sigma-Aldrich) and diammonium hydrogen phosphate ((NH_4_)_2_∙HPO_4_, Sigma-Aldrich) was dissolved in distilled water by vigorously stirring at a rate of 1000 rpm. The pH of the mixed solution was maintained at 8 and 11 by the addition of ammonium hydroxide (NH_4_OH, Junsei) solution. The co-precipitated suspension was discharged from the reactor and allowed to settle for 24 h for the maturation of precipitate. After 24 h, the precipitates were separated through vacuum filtration technique and dried at 80 °C for 24 h in a drying oven. The as-dried precipitates were calcined at 1000 °C for 24 h in air.

X-ray diffraction analysis (X’Pert Pro, Philips), at 40 kV and 40 mA with a scanning speed of 1o/min, was performed to identify the phases of the as-calcined powders. A standard Bragg-Brentano geometry was applied with a K_α_1 monochromatic beam from the Cu anode. Phase identification, quantitative analysis, determination of Ca/P ratio, and lattice parameters for the BCP powders were characterized using Phillips X’Pert HighScore Plus software with a full-pattern fit using Rietveld method [17, 18]. The Raman spectra were recorded on a Sentinel Raman spectrometer (Bruker Optics Ltd.) with a Unilab II probe (fiber optic) and a CCD detector was used in this study. A 532 nm Nd: YAG laser source was used for excitation with an incident laser power of 30 mW. The spectral range was 500 to 4400 cm^−1^ with a resolution of 4 to 6 cm^−1^.

## Results and discussion

**Fig. 1:**
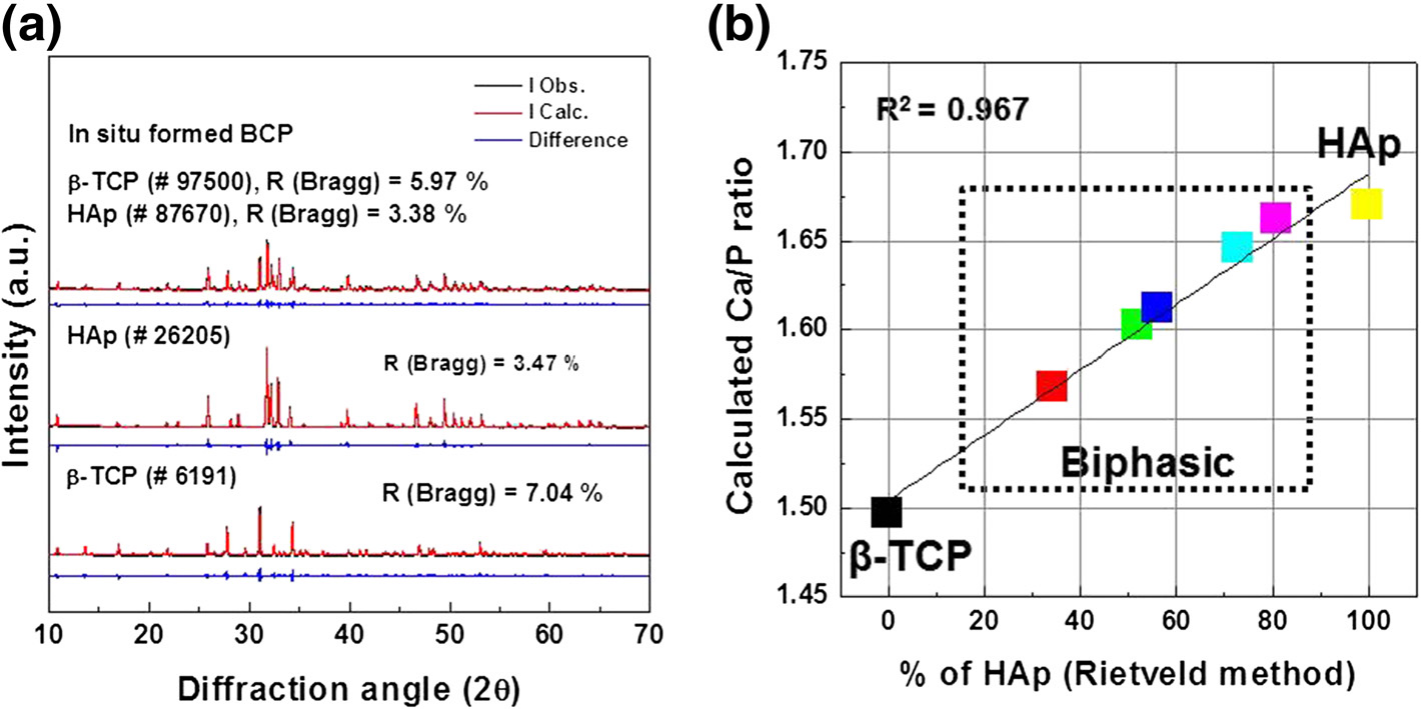
**a** Rietveld analysis patterns of powder diffraction data of BCP and **b** molar Ca/P ratio of BCP calculated using Rietveld method

Figure 1 shows Rietveld analysis, determined crystal phase %, and calculated Ca/P ratio for as-synthesized β-TCP, HAp, and BCP powders. Figure 1(a) shows fitting deviations of XRD patterns of β-TCP, HAp, and BCP powders with Rietveld analysis. Pattern fitting was carried out between 10 and 70°. As shown in Fig. 1(a), Rietveld refinement was performed using the structural model of ICSD card number # 6191 and 97500 for β-TCP phase. The structural model of ICSD card number # 26205 and 87670 were used for HAp phase. Figure 1(b) shows the percentage of relative determined crystal phase and calculated Ca/P ratio of the β-TCP, HAp, and BCP powders through Rietveld analysis. An attempt was also made to determine the reliability of the resultant phase mixtures obtained through refinement with those of experimental Ca/P ratio of the precursors according to Equation (2):

2 $$ \mathrm{Expected}\ \mathrm{C}\mathrm{a}/\mathrm{P} = \mathrm{wt}\ \%\ \mathrm{of}\ \mathrm{H}\mathrm{A}\mathrm{p}\ \left(\mathrm{Rietveld}\right)*\ 1.67 + \mathrm{wt}\ \%\ \mathrm{of}\ \mathrm{T}\mathrm{C}\mathrm{P}\ \left(\mathrm{Rietveld}\right)\ *\ 1.5 $$

As shown in Fig. 1(b), BCP powders have indicated only the presence of HAp and β-TCP in its composition but their quantitative phase contents determined through Rietveld analysis were found to show significant variations, and their Ca/P ratio were totally dependent on the percentage of determined crystal phase.

**Fig. 2:**
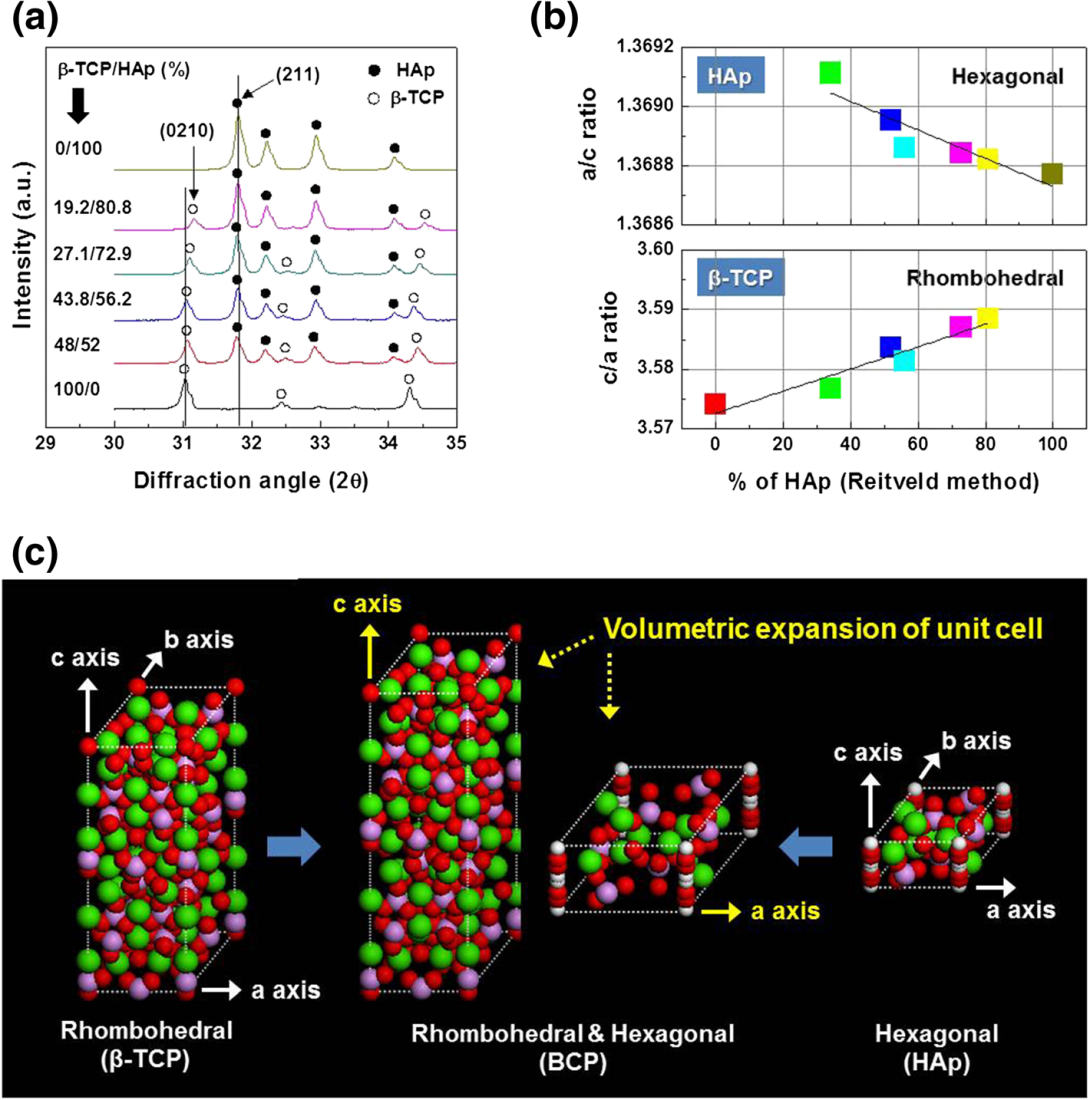
**a** XRD patterns, **b** axial ratio, and **c** volumetric expansion behavior of unit cells of *in-situ* formed BCP. (Note: axis of β-TCP and HAp is a = b and axial angle is α = β = 90°, γ = 120°)

Figure 2 shows biphasic behaviors and effects of crystal system of in situ formed BCP with different phase contents. The expanded XRD patterns of BCP in the close scan around the main peak region (2θ = 30-35°) as shown in Fig. 2(a). The β-TCP (0210) peak is well distinguished and differentiated from the HAp (211) peak. Figure 2(b) shows axis ratio of β-TCP and HAp phase in the lattice parameters of *in-situ* formed BCP with different phase contents. In the case of *in-situ* formed BCP, the as-calculated c/a axis ratio of β-TCP phase was increased with increasing HAp phase contents as shown in Fig. 2(b). On the other hand, the a/c axis ratio of HAp showed a little decrease to closed a/c ratio of monophasic HAp. In contrast to result of β-TCP, the as-calculated a/c axis ratio of HAp phase of *in-situ* formed BCP was decreased to the theoretical a/c axis ratio (a/c ratio = 1.3689, ICSD # 87670). In addition, a volume for unit cell of *in-situ* formed BCP showed the volumetric expansion, compared to the monophasic HAp and β-TCP, as shown in Fig. 2(c).

**Fig. 3:**
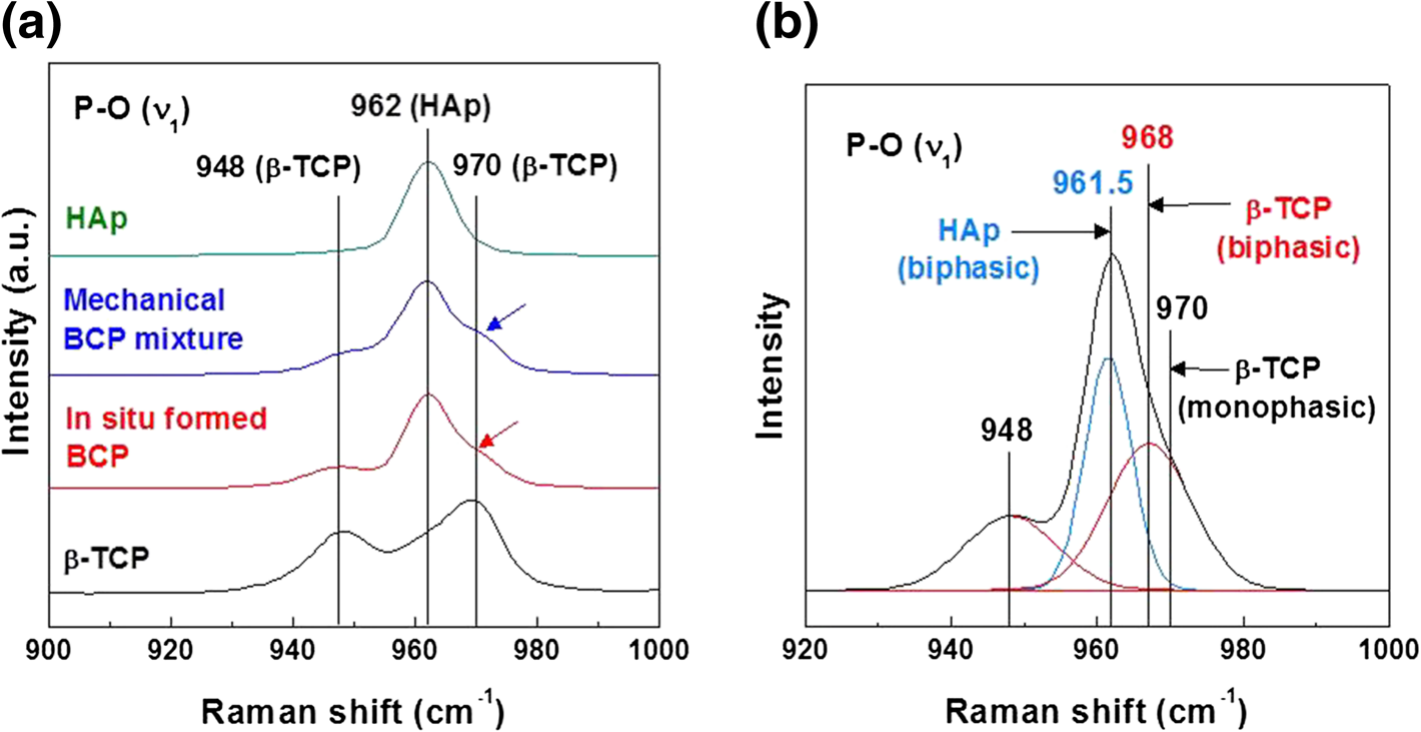
**a** Symmetric vibration (stretching mode, ν_1_) of PO_3_^4−^ of β-TCP, HAp, mechanical mixed BCP, and *in-situ* formed BCP and **b** pseudo Gaussian fitting results from Raman spectra of *in-situ* formed BCP

Figure 3 shows the short range order structural analysis of *in-situ* formed BCP. Figure 3(a) shows the Raman spectra of β-TCP, HAp, mechanical mixed BCP, and *in-situ* formed BCP, assigned to a symmetric P-O vibration (stretching mode, ν_1_) within the PO_3_^4−^ group. Among the spectra shown in Fig. 3(a), it is spectrum of the *in-situ* formed BCP that is most similar to spectra observed from mixture of pure β-TCP and HAp. However, it is evident that P-O vibration peak (i.e., 968 cm^−1^ of β-TCP) of *in-situ* formed BCP (i.e., 43.8 % of β-TCP and 56.2 % of HAp determined by Rietveld analysis) indicated a peak shift, compared with monophasic β-TCP and mechanical mixed BCP (i.e., 44 % of β-TCP and 56 % of HAp), as shown in Fig. 3(b). Therefore, the short range order structure of *in-situ* formed BCP could be considered to be affected by the coexistence of β-TCP and HAp phase.

## Conclusions

HAp, β-TCP and in situ formed BCP powders were synthesized by the co-precipitation and calcination process. The refined structural parameters of *in-situ* formed BCP confirmed that all the investigated structures have crystallized in the corresponding hexagonal (space group P63/m) and rhombohedral (space group R3c) structures. The molar Ca/P ratio of *in-situ* formed BCP was also determined by Rietveld analysis. The crystal phases, Ca/P molar ratio, and lattice parameter of *in-situ* formed BCP consisting of HAp and β-TCP were controlled by the degree of calcium deficiency of calcium phosphate precursors. The significant short range order structural change of BCP was determined by Raman analysis. The short range order structures of *in-situ* formed BCP was considered to be affected by the coexistence of β-TCP and HAp phase.

## Availability of supporting data

The data set supporting the results of this article is included within the article Tables 1, 2, 3, 4 are represented structure model of HAp and β-TCP crystal in XRD results of this study (Additional file 1).

## Additional file

Additional file 1: Table S1. Structure model of HAp (ICSD card number: # 26205). **Table S2.** Structure model ofHAp (ICSD card number: # 87670). **Table S3.** Structure model of β-TCP (ICSD card number: # 6191). **Table S4.** Structure model of β-TCP (ICSD card number: # 97500).
