# Supplementary material for: Long and short range order structural analysis of In-situ formed biphasic calcium phosphates
Source: Biomater Res. 2015 Dec 31;19:14. doi: 10.1186/s40824-015-0036-0 (PMC4697325; doi:10.1186/s40824-015-0036-0)
Supplement: Additional file 1: Table S1. — Structure model of HAp (ICSD card number: # 26205). Table S2. Structure model ofHAp (ICSD card number: # 87670). Table S3. Structure model of β-TCP (ICSD card number: # 6191). Table S4. Structure model of β-TCP (ICSD card number: # 97500). [file 40824_2015_36_MOESM1_ESM.docx]

**Availability of supporting data**

The data set supporting the results of this article is included within the article.

Table 1, 2, 3, 4 are represented structure model of HAp and β-TCP crystal in XRD results of this study.

**Table 1** Structure model of HAp (ICSD card number: # 26205).

| **No.** | **Name** | **Elem.** | **X** | **Y** | **Z** | **Biso** | **sof** | **Wyck.** |
| --- | --- | --- | --- | --- | --- | --- | --- | --- |
| 1 | H1 | H | 0 | 0 | 0.0608 | 0.5 | 0.5 | 4e |
| 2 | O1 | O | 0 | 0 | 0.1978 | 0.5 | 0.5 | 4e |
| 3 | CA1 | Ca | 0.2465 | 0.2534 | 0.25 | 0.5 | 1 | 6h |
| 4 | CA2 | Ca | 0.33333 | 0.66667 | 0.0013 | 0.5 | 1 | 4f |
| 5 | P1 | P | 0.3983 | 0.03 | 0.25 | 0.5 | 1 | 6h |
| 6 | O2 | O | 0.3433 | 0.0854 | 0.0704 | 0.5 | 1 | 12i |
| 7 | O3 | O | 0.5876 | 0.1224 | 0.25 | 0.5 | 1 | 6h |
| 8 | O4 | O | 0.1564 | 0.4846 | 0.25 | 0.5 | 1 | 6h |

**Table 2** Structure model of HAp (ICSD card number: # 87670).

| **No.** | **Name** | **Elem.** | **X** | **Y** | **Z** | **Biso** | **sof** | **Wyck.** |
| --- | --- | --- | --- | --- | --- | --- | --- | --- |
| 1 | H1 | H | 0 | 0 | 0.0608 | 0.5 | 0.937 | 4e |
| 2 | O1 | O | 0 | 0 | 0.1999 | 0.5 | 0.553 | 4e |
| 3 | O2 | O | 0.3424 | 0.0847 | 0.0685 | 0.5 | 1 | 12i |
| 4 | O3 | O | 0.586 | 0.1211 | 0.25 | 0.5 | 1 | 6h |
| 5 | O4 | O | 0.1569 | 0.4844 | 0.25 | 0.5 | 1 | 6h |
| 6 | P1 | P | 0.3979 | 0.0301 | 0.25 | 0.5 | 0.993 | 6h |
| 7 | CA1 | Ca | 0.2467 | 0.2534 | 0.25 | 0.5 | 1 | 6h |
| 8 | CA2 | Ca | 0.33333 | 0.66667 | 0.0017 | 0.5 | 1 | 4f |

**Table 3** Structure model of β-TCP (ICSD card number: # 6191).

| **No.** | **Name** | **Elem.** | **X** | **Y** | **Z** | **Biso** | **sof** | **Wyck.** |
| --- | --- | --- | --- | --- | --- | --- | --- | --- |
| 1 | O1 | O | 0.2302 | 0.2171 | 0.1846 | 0.5 | 1 | 18b |
| 2 | CA1 | Ca | 0.15583 | 0.28307 | 0.24027 | 0.5 | 1 | 18b |
| 3 | CA2 | Ca | 0.18513 | 0.39457 | 0.14607 | 0.5 | 1 | 18b |
| 4 | CA3 | Ca | 0 | 0 | 0.125 | 0.5 | 0.491 | 6a |
| 5 | CA4 | Ca | 0 | 0 | 0.3058 | 0.5 | 0.999 | 6a |
| 6 | P1 | P | 0 | 0 | 0.04 | 0.5 | 1 | 6a |
| 7 | O2 | O | 0.1436 | 0.007 | 0.0536 | 0.5 | 1 | 18b |
| 8 | O3 | O | 0 | 0 | 0 | 0.5 | 1 | 6a |
| 9 | CA5 | Ca | 0.19123 | 0.39007 | 0.04087 | 0.5 | 1 | 18b |
| 10 | O4 | O | 0.2736 | 0.09 | 0.1326 | 0.5 | 1 | 18b |
| 11 | O5 | O | 0.0053 | 0.2788 | 0.1923 | 0.5 | 1 | 18b |
| 12 | O6 | O | 0.09413 | 0.18897 | 0.34447 | 0.5 | 1 | 18b |
| 13 | P2 | P | 0.3465 | 0.1537 | 0.2733 | 0.5 | 1 | 18b |
| 14 | O7 | O | 0.4031 | 0.0489 | 0.2611 | 0.5 | 1 | 18b |
| 15 | O8 | O | 0.09127 | 0.45233 | 0.08853 | 0.5 | 1 | 18b |
| 16 | O9 | O | 0.1814 | 0.0805 | 0.2633 | 0.5 | 1 | 18b |
| 17 | O10 | O | 0.3696 | 0.1748 | 0.3135 | 0.5 | 1 | 18b |
| 18 | P3 | P | 0.3109 | 0.1365 | 0.172 | 0.5 | 1 | 18b |

**Table 4** Structure model of β-TCP (ICSD card number: # 97500).

| **No.** | **Name** | **Elem.** | **X** | **Y** | **Z** | **Biso** | **sof** | **Wyck.** |
| --- | --- | --- | --- | --- | --- | --- | --- | --- |
| 1 | O1 | O | 0.2326 | 0.2167 | 0.1873 | 1.66 | 1 | 18b |
| 2 | CA1 | Ca | 0.15883 | 0.28547 | 0.24197 | 0.27 | 1 | 18b |
| 3 | CA2 | Ca | 0.18473 | 0.39327 | 0.14767 | 0.76 | 1 | 18b |
| 4 | CA3 | Ca | 0 | 0 | 0.1272 | 2 | 0.43 | 6a |
| 5 | CA4 | Ca | 0 | 0 | 0.3085 | 0.76 | 1 | 6a |
| 6 | P1 | P | 0 | 0 | 0.0421 | 0.41 | 1 | 6a |
| 7 | P2 | P | 0.3128 | 0.1394 | 0.1736 | 0.29 | 1 | 18b |
| 8 | CA5 | Ca | 0.19513 | 0.39257 | 0.04247 | 0.38 | 1 | 18b |
| 9 | O2 | O | 0.2744 | 0.0944 | 0.1338 | 1.79 | 1 | 18b |
| 10 | O3 | O | 0 | 0 | 0 | 1.06 | 1 | 6a |
| 11 | O4 | O | 0.0088 | 0.279 | 0.1935 | 0.77 | 1 | 18b |
| 12 | O5 | O | 0.09413 | 0.18877 | 0.34607 | 1.25 | 1 | 18b |
| 13 | O6 | O | 0.4013 | 0.0488 | 0.2627 | 0.44 | 1 | 18b |
| 14 | O7 | O | 0.09287 | 0.45253 | 0.09043 | 1.32 | 1 | 18b |
| 15 | O8 | O | 0.1813 | 0.0803 | 0.265 | 0.27 | 1 | 18b |
| 16 | O9 | O | 0.368 | 0.1742 | 0.3153 | 0.84 | 1 | 18b |
| 17 | O10 | O | 0.1433 | 0.0057 | 0.0536 | 1.36 | 1 | 18b |
| 18 | P3 | P | 0.347 | 0.1536 | 0.2753 | 0.14 | 1 | 18b |
